# Chromosomal gene order defines several structural classes of *Staphylococcus epidermidis* genomes

**DOI:** 10.1371/journal.pone.0311520

**Published:** 2024-10-04

**Authors:** Naya Nagy, Paul Hodor

**Affiliations:** 1 College of Computer Science and Information Technology, Imam Abdulrahman Bin Faisal University, Dammam, Saudi Arabia; 2 Aurynia LLC, Seattle, Washington, United States of America; Academia Sinica, TAIWAN

## Abstract

The original methodology for describing the pangenome of a prokaryotic species is based on modeling genomes as unordered sets of genes. More recent findings have underlined the importance of considering the ordering of genes along the genetic material as well, when making comparisons among genomes. To further investigate the benefits of gene order when describing genomes of a given species, we applied two distance metrics on a dataset of 84 genomes of *Staphylococcus epidermidis*. The first metric, GeLev, depends on the order of genes and is a derivative of the Levenshtein distance. The second, the Jaccard distance, depends on gene sets only. The application of these distances reveals information about the *global* structure of the genomes, and allows clustering of the genomes into classes. The main biological result is that, while genomes within the same class are structurally similar, genomes of different classes have an additional characteristic. Between genomes in different classes we can discover instances where a large *segment* of the first genome appears in reverse order in the second. This feature suggests that genome rearrangements in *S. epidermidis* happen on a large scale, while micro-rearrangements of single or a small number of genes are rare. Thus, this paper describes a straight-forward method to classify genomes into structural classes with the same order of genes and makes it possible to visualize reversed segments in pairs of genomes. The method can be readily applied to other species.

## Introduction

Examination of the genomes of multiple strains from the same prokaryotic species has led to the concept of the pangenome, which describes the gene repertoire of a species and the frequency of genes among strains [1, 2]. The pangenome is operationally divided into *core genes*, which occur in every strain, *accessory or dispensable genes*, which occur in some, but not all strains, and *strain-specific genes*, which occur in one or very few strains. The exact composition of these gene sets depends on the number and diversity of analyzed genomes. This approach has proven to be a useful tool in comparative genomics of prokaryotes and has been applied to a large variety of species and other taxonomic levels [3, 4].

In the original pangenomic approach, genomes were modeled as sets of genes without taking into consideration the linear order in which genes are arranged on the chromosome. It is well-known that genes are not distributed randomly along the chromosome, and that their order may have functional and/or evolutionary significance. For example, Sonnenberg et al. [5] considered the distance of genes from the origin of replication in *Vibrionaceae* and found that core genes are more closely associated with it than accessory genes.

Genomic distance metrics, which consider gene (or other DNA sequence marker) order, have been discussed in the literature for over 25 years. Sankoff [6] considered breakpoints, i.e. points in the genome where the order of genes does not match, and described genomic distance based on the minimum number of rearrangements, additions, deletions, and inversions, to generate one genome from the other. Hannenhalli and Pevzner [7] developed an algorithm for computing the “reversal distance” between two genomes, where each genome is considered as a permutation of genes.

These classic results have two limitations: First, there is no definitive method to assign homologous genes, leading to the possibility of ambiguous and/or replicated gene markers. Second, algorithms for computing inter-genome distances that rely on evolutionary models need to take into account lateral gene transfer, which is a common process in prokaryotes. Newer studies have addressed these issues in different ways.

The following, more recent research outputs, alleviate the first limitation of homologous genes. Bohnenkämper et al. [8] improved the algorithm for computing distances in the presence of gene duplications, while Rubert et al. [9] developed a genomic distance based on pairwise similarities of DNA fragments, without the need of assigning genes to families.

The second limitation was addressed by a different distance metric, the synteny index, described by Shifman et al. [10]. It is based on local similarities in gene content and was shown to be robust to horizontal gene transfer [11]. Another distance estimation based on gene order was proposed by House et al. [12]. It uses Monte Carlo sampling of 5-6 orthologs and computes a metric that depends on whether or not the selected genes were in the same order.

Attempts to focus on small regions of DNA can be found in the graph representations of Urhan et al. [13]. These graphs compare 70 genomes of *Acinetobacter baumannii* produced by a large variety of pangenome construction tools and show structural variation due to transposons and variations in local context of plasmid genes.

There are several recent examples of pangenome analysis and visualization tools, which incorporate syntenic properties of prokaryotic genomes. Pan-Explorer [14] provides several graphical representations of genomes, and in particular, synteny information is represented as Hive plots for global visualisations and as Mauve views for zoom ins. The comparison is restricted to three genomes. PPanGGOLiN [15, 16] is capable of analyzing genomes on a large scale. The graph visualizations are done with the Gephi software. Graphs contain gene families as nodes and weighted edges show colocalization. The graphs can be overwhelming in the amount of data that they visualize. Panakeia [17] uses gene clustering to provide synteny information between groups. As such, members of each cluster need be similar down to 70%. Graph edges show proximity of groups. Panakeia is able to aggregate synteny information on hundreds of prokaryotic genomes including their plasmids. Panakeia helps in the study of both vertical and horizontal transfer of genes. Sibelia [18, 19] does synteny analysis as a hierarchy of increasing gene blocks. It efficiently builds de Bruijn graphs, running several times faster than comparable tools. An alternative algorithm based on colored de Bruijn graphs is described by Schulz et al. [20]. It is used to detect the pangenome core and has the advantage of a small memory footprint, making it applicable to both prokaryotes and eukaryotes.

In the present study we sought to develop a new distance metric to compare closely related prokaryotic genomes. The desired properties of the metric are as follows:

1. The distance should not be based on a specific model of phylogenetic evolution. This circumvents the difficulty of balancing vertical vs. horizontal gene transfer. More importantly, it focuses the interpretation of results on direct relationships among considered genomes, rather than their evolutionary history.
2. It should include information on both gene repertoire and order. The combination of repertoire and order would enhance the traditional pangenomic approach based on repertoire alone with important information, as described above.
3. Computation of the distance should be simple and deterministic. This would allow robust reproducibility of results.

As our distance metric for this study, we chose an adaptation of the Levenshtein string edit distance. Genes forming the pangenome were considered as the alphabet, and each genome, an ordered string of genes. Genome relationships discovered with this approach were compared with relationships based on pangenomic gene sets alone.

To test our approach, we chose the gram-positive bacterium *Staphylococcus epidermidis* as a model organism. *S. epidermidis* is a common commensal found on mammalian skin and the most frequently isolated species from human epithelia [21]. Even though typically benign, it often causes infections in humans. In fact, *S. epidermidis* is the major cause of hospital infections by foreign objects, such as catheters, joint prostheses, and CSF shunts [22, 23]. Previous comparison of *S. epidermidis* genomes by Conlan et al. [24] uncovered two major clusters of strains defined by sequence similarity of core genes, in which strains that were either commensals or infection-associated were unevenly distributed. Clustering based on gene content was not performed, and gene order was considered only in the case of the neighborhood of the formate dehydrogenase gene.

## Methods

### Overview

**Fig 1:**
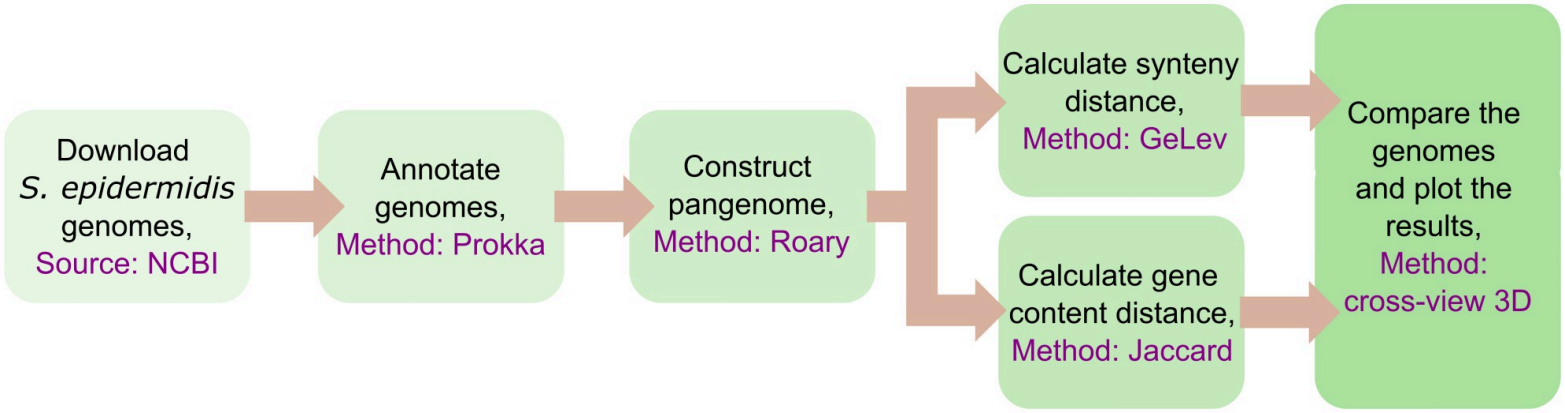
Method overview. *S. epidermidis* genomes were downloaded, annotated, and used to construct a pangenome. Distance matrices based on gene order or unordered gene composition were computed and used for downstream analysis.

The broad analytical steps taken in this study are outlined in Fig 1. Genome sequences of *S. epidermidis* were downloaded from the National Center for Biotechnology Information (NCBI). They were annotated with open reading frame information using the software Prokka. A pangenome was constructed using Roary. Two types of distance matrices were computed: 1. GeLev, based on gene order, and 2. Jaccard, based on unordered gene sets. Finally, the results were analyzed and plotted in R. Details of each step are given below.

### Sequence data

Nucleotide sequences of *S. epidermidis* strains were downloaded by FTP from the Genome resource at NCBI [25]. Only complete genomes were selected. As of early 2022, there were 84 complete genomes available (Table 1).

**Table 1:** 84 *S. epidermidis* genomes used in this study. They are divided into 10 clusters, with one genome selected as representative (bold), as described in the Results section. Individual genomes are numbered within their cluster in alphabetical order of their chromosome accession numbers. The third, fourth and fifth column identify the genome.

Cluster	Index within Cluster	Chromosome Accession Number	Assembly	Strain
L1	1	NZ_CP073821.1	ASM1932932v1	B1200343
	2	NZ_CP073824.1	ASM1932924v1	B1200599
	3	NZ_CP073827.1	ASM1932936v1	B1208538
	4	NZ_CP073830.1	ASM1932940v1	B1220165
	5	NZ_CP073835.1	ASM1932942v1	B1230143
	6	NZ_CP073836.1	ASM1932950v1	B1262351
	7	NZ_CP073840.1	ASM1932952v1	B1264454
	8	NZ_CP073841.1	ASM1932954v1	B1264787
	9	NZ_CP073844.1	ASM1932956v1	B1265603
	**10**	**NZ_CP073847.1**	**ASM1932958v1**	**B1266875**
	11	NZ_CP073850.1	ASM1932960v1	B1266911
	12	NZ_CP073852.1	ASM1932962v1	B1266915
	13	NZ_CP073855.1	ASM1932964v1	B1269488
	14	NZ_CP073857.1	ASM1932966v1	B1272014
	15	NZ_CP073859.1	ASM1932968v1	B1275857
	16	NZ_CP073862.1	ASM1932970v1	B1276219
	17	NZ_CP073863.1	ASM1932972v1	B1276296
	18	NZ_CP073865.1	ASM1932974v1	B1276912
	19	NZ_CP073869.1	ASM1932976v1	B1279591
	20	NZ_CP073872.1	ASM1932978v1	B1282731
	21	NZ_CP073876.1	ASM1932980v1	B1285135
	22	NZ_CP073878.1	ASM1978766v1	B1285228
	23	NZ_CP073881.1	ASM1933004v1	B1286247
	24	NZ_CP073883.1	ASM1933010v1	B1287175
	25	NZ_CP073887.1	ASM1933012v1	V1933625
	26	NZ_CP073890.1	ASM1933014v1	V1933793
	27	NZ_CP073893.1	ASM1933016v1	V1936703
	28	NZ_CP073895.1	ASM1933018v1	V1937538
	29	NZ_CP073898.1	ASM1933020v1	V1939586
	30	NZ_CP073900.1	ASM1933022v1	V1949610
	31	NZ_CP073904.1	ASM1933024v1	V1950266
	32	NZ_LR134536.1	59178_A02	NCTC13924
L2	1	NZ_AP019721.1	ASM674220v1	NBRC 100911
	2	NZ_CP009046.1	ASM75955v1	SEI
	3	NZ_CP014119.1	ASM294499v1	FDAARGOS_153
	4	NZ_CP014132.1	ASM295405v1	FDAARGOS_161
	5	NZ_CP020463.1	ASM208569v1	1457
	6	NZ_CP030246.1	ASM332573v1	CSF41498
	7	NZ_CP034111.1	ASM385639v1	CDC120
	8	NZ_CP034115.1	ASM385645v1	CDC121
	9	NZ_CP035288.1	ASM609437v1	ATCC 14990
	10	NZ_CP035643.1	ASM1103857v1	E73
	11	NZ_CP043847.1	ASM976912v1	NCCP 16828
	12	NZ_CP060528.1	ASM1433429v1	LM087
	13	NZ_CP060794.1	ASM1448933v1	Z0118SE0260
	14	NZ_CP066376.1	ASM1640662v1	PH1-28
	15	NZ_CP068136.1	ASM1672738v1	FDAARGOS_1083
	**16**	**NZ_CP069215.1**	**ASM1683447v1**	**Z0118SE0269**
	17	NZ_CP069219.1	ASM1683449v1	Z0118SE0272
	18	NZ_CP069473.1	ASM1688916v1	FDAARGOS_1243
	19	NZ_CP069951.1	ASM1690355v1	FDAARGOS_1363
	20	NZ_CP070057.1	ASM1690413v1	FDAARGOS_1361
	21	NZ_CP071988.1	ASM2139830v1	CBPA-ST-10002
	22	NZ_CP071994.1	ASM2139834v1	AZ22
	23	NZ_CP084008.1	ASM2018139v1	NBRC 113846
	24	NZ_CP090575.1	ASM2148486v1	11H
	25	NZ_CP090985.1	ASM2151307v1	50D
	26	NZ_CP090989.1	ASM2151305v1	52B
	27	NZ_CP090993.1	ASM2151311v1	BC1190
	28	NZ_CP090998.1	ASM2151309v1	BC1191
	29	NZ_CP091006.1	ASM2151303v1	R10C
	30	NZ_LR735421.1	Se_BPH0697	Se_BPH0697
	31	NZ_LR735437.1	Se_BPH0723	Se_BPH0723
	32	NZ_LR735440.1	Se_BPH0711	Se_BPH0711
M1	1	NZ_CP018842.1	ASM195665v2	14.1.R1
	2	NZ_CP022247.1	ASM221553v1	AMT
	3	NZ_CP033782.1	ASM381242v1	FDAARGOS_529
	**4**	**NZ_CP061029.1**	**ASM1449051v1**	**Z0118SE0132**
	5	NZ_CP071992.1	ASM2139832v1	CBPA-ST-11003
	6	NZ_LR735429.1	Se_BPH0704	Se_BPH0704
M2	**1**	**NC_002976.3**	**ASM1192v1**	**RP62A**
	2	NZ_CP040883.1	ASM1331712v1	O47
	3	NZ_LR735432.1	Se_RP62a_UoM	Se_RP62a-WT
	4	NZ_LR735434.1	Se_BPH0736	Se_BPH0736
M3	**1**	**NC_004461.1**	**ASM764v1**	**ATCC 12228**
	2	NZ_CP043845.1	ASM987345v1	ATCC 12228
	3	NZ_CP065656.1	ASM1602691v1	FDAARGOS_913
M4	1	NZ_CP013943.1	ASM285031v1	DAR1907
	2	NZ_CP045648.1	ASM968513v1	IRL01
	**3**	**NZ_LT571449.1**	**BPH0662**	**BPH0662**
S1	**1**	**NZ_CP069954.1**	**ASM1690357v1**	**FDAARGOS_1364**
S2	**1**	**NZ_CP043841.1**	**ASM976901v1**	**NCCP 16829**
S3	**1**	**NZ_HG813242.1**	**PM221**	**PM221**
S4	**1**	**NZ_CP066303.1**	**ASM1640622v1**	**SE48**

### Genome annotation

Genome annotation was done with the software Prokka 1.14.5 [26], with default parameters. The output of Prokka consisted of one annotation file in GFF format for each genome. Each file contained a list of all coding sequences and RNA genes found on the chromosome and any plasmids. For each gene, the following information was output:

1. the beginning and end position within the sequence,
2. the name of the gene, if it is a known gene, and
3. the direction in which the gene is to be read.

Downstream analysis was restricted to chromosomal coding sequences, i.e. plasmid sequences and tRNA and rRNA genes were excluded.

Note that the output of Prokka had a couple of limitations that had to be addressed in downstream analysis. First, *S. epidermidis* has a circular chromosome, but GFF sequence positions were represented linearly. Position 1 was located arbitrarily in different input sequences, depending on where the chromosome was conceptually cut open. Thus the best match of gene order among strains had to involve some shift in the beginning position and potentially taking the reverse complement of the entire chromosome. Second, Prokka assigned gene names independently for each input sequence, meaning that the same gene in different strains usually had inconsistent names.

### Pangenome construction

Construction of the pangenome was done with Roary 3.13.0 [27, 28]. The input to Roary were the GFF files produced by Prokka. Roary recognized identical genes from within the entire population of strains and gave them unique names. Therefore, a comparison could be made as to whether a specific gene existed in a genome from within the set of genomes under scrutiny. For example, the gene names given by Prokka: *FEAOMMGP_01120* and *OGLCDFDM_01020*, were recognized as representing the same gene and Roary gave the name *nudF*.

Roary was run in parallel with 64 CPUs on an m4.16xlarge instance in Amazon Web Services (AWS). The output contained a file in CSV format showing presence/absence calls for each gene across the 84 input genomes. We considered core genes those that were present in all 84 genomes and accessory genes those in at least 35% of genomes (30 genomes).

### Distance calculation

Two distance metrics were defined: GeLev and Jaccard (see the following sections for details). For each distance, all pairwise distances were calculated across the collection of genomes and output as a distance matrix. Custom programs were written in C++ and compiled with g++ 12.1.0 (https://gcc.gnu.org) and the boost library (https://www.boost.org). For GeLev the genomes were represented as arrays of genes and for Jaccard as sets. The GeLev calculation was implemented with a dynamic programming algorithm that had quadratic complexity. It took about 1/2 hour to compute the GeLev distance matrix on a 128 CPU type c6i.32xlarge instance in AWS. The Jaccard calculation had linear asymptotic complexity in the size of the genomes and was very quick on a minimal computing instance.

### Analysis of results

The analysis and interpretation of results was done using R 4.2.3 [29]. Additional packages included ape 5.7.1 [30] used for parsing GFF files and construction of phylogenetic trees and genoPlotR 0.8.11 [31] for visualizations of genome alignments.

### Normalized Levenshtein distance, GeLev

The **Levenshtein distance** was initially developed for and applied to the transmission of binary information as bits over a lossy channel. Within the transmitted array of bits, faults can appear. Bits can be swapped, added, or deleted, while the binary information must still be fully retrievable [32].

More recently, the same distance metric has proven to be useful to compare DNA or RNA sequences based on differences in bases [33]. In this case, the alphabet consisted of four letters, the bases of the nucleic acids. The Levenshtein distance was used to improve similarity search between sequences.

In general, the Levenshtein distance compares two *s*trings of letters defined over an alphabet. The distance takes into consideration the ordered string, such that the arrayed elements contribute constructively or destructively to the value of the distance. The comparison is done by counting the number of deletions, insertions, and substitutions.

In our present study, the Levenshtein distance was used to compare entire genomes based on their gene content. Each genome was modeled as a string, where a letter represented a single gene. Thus, the alphabet consisted of all the genes that appeared in the pangenome, making the size of the alphabet very large. Another characteristic of this study was that gene duplications in prokayotic genomes are rare, and therefore, typically, any letter appeared at most once in the strings to be compared.

The following explains the operations that contribute to the Levenshtein distance, as applied in this study.

1. **Insertion** is the operation where a gene does not appear in the first genome, but appears in the second. For example, if the first genome has the genes *Genome*_1_ = …, *gene*_*A*_, *gene*_*B*_, *gene*_*C*_, *gene*_*D*_, …., and the second genome is *Genome*_2_ = …, *gene*_*A*_, *gene*_*B*_, *gene*_*X*_, *gene*_*C*_, *gene*_*D*_, …, then there was an insertion of *gene*_*X*_ into the second genome, and the Levenshtein distance increases by 1.
2. **Deletion** is the reverse operation of **Insertion**. A gene appears in the first genome, but is removed from within the sequence of the second genome. For example, for *Genome*_1_ = …, *gene*_*A*_, *gene*_*B*_, *gene*_*C*_, …, and *Genome*_2_ = …, *gene*_*A*_, *gene*_*C*_, …, the gene *gene*_*B*_ was deleted, and the Levenshtein distance increases by 1.
3. **Substitution** is an operation in which one gene of the first genome is replaced by another. For example, for *Genome*_1_ = …, *gene*_*A*_, *gene*_*B*_, *gene*_*C*_, …, and *Genome*_2_ = …, *gene*_*A*_, *gene*_*X*_, *gene*_*C*_, …, the *gene*_*B*_ was replaced by *gene*_*X*_. Even though, this operation seems more involved as the first two, we consider this change to also contribute with an addition of 1. Note that some authors in the literature consider the substitution operation as contributing with a value of 2, in which case, substitution is considered to be a deletion, followed by an insertion. In our model, we chose the penalty of 1, as our GeLev is strictly based on order.

As can be seen from the definitions above, in the case of two identical genomes, the Levenshtein distance is 0. In the following formulas, the length of the genome is defined as the number of genes that appear in the genome. For genomes of non-equal length, there is a simple way to evaluate the maximum and minimum of the Levenshtein distance. For genomes *G*_1_ and *G*_2_ of lengths ||*G*_1_|| = *m* and ||*G*_2_|| = *n*, the minimum Levenshtein distance is the extra genes in the longer genome, namely *min*(*Lev*(*G*_1_, *G*_2_)) = |*m* − *n*|. The maximum Levenshtein distance is given by the larger genome, if the two genomes have no common genes. Thus, *max*(*Lev*(*G*_1_, *G*_2_)) = *max*(*m*, *n*).

As can be seen from the definition above, the Levenshtein distance is upper bounded by a value that depends on the length of the strings to be compared. Therefore, the Levenshtein distance depends on the length of the strings themselves and cannot be used as a universal measure of order similarity for arbitrary string lengths. It would be beneficial to normalize the Levenshtein distance. In this case, all values would be enclosed in the interval between 0 and 1, and the meaning of the distance would be independent of the length of the string. For a normalized Levenshtein distance, 0 means identical strings and 1 means strings with maximum difference.

We define a metric for the purpose of our research, to be called **Ge**ne Content Normalized **Lev**enshtein Distance (GeLev). The GeLev metric is emphasizing, or measuring, the order of the genes. Thus two genomes that have different lengths, but have all common genes in the same order, are a perfect match. The GeLev in this case has to evaluate to zero. By contrast, two genomes that contain totally different genes, have a GeLev of one. For two genomes *G*_1_ and *G*_2_, denote *Lev*(*G*_1_, *G*_2_) the original, not-normalized Levenshtein distance. Then the GeLev distance is defined by the formula

$$\begin{array}{c} GeLev\left(G_{1},G_{2}\right)=\frac{Lev\left(G_{1},G_{2}\right)-\left|g_{1}-g_{2}\right|}{max\left(g_{1},g_{2}\right)-\left|g_{1}-g_{2}\right|} \end{array}$$ (1)

In the above formula, we denoted the lengths of the genomes with their lower case letters, ||*G*_1_|| = *g*_1_ and ||*G*_2_|| = *g*_2_. |*g*_1_ − *g*_2_| means the difference in length between the two genomes, and *max*(*g*_1_, *g*_2_) means the length of the longer genome. Additionally, we can see that the denominator actually represents the shorter genome, *max*(*g*_1_, *g*_2_) − |*g*_1_ − *g*_2_| = *min*(*g*_1_, *g*_2_). Thus, the formula of the GeLev can be rewritten as

$$\begin{array}{c} GeLev\left(G_{1},G_{2}\right)=\frac{Lev\left(G_{1},G_{2}\right)-\left|g_{1}-g_{2}\right|}{min(g_{1},g_{2})} \end{array}$$ (2)

Now, to check the validity of the metric at the extremes, we have the following:

- When the genomes are identical and have the same length, then *Lev*(*G*_1_, *G*_2_) = 0, |*g*_1_ − *g*_2_| = 0, and *min*(*g*_1_, *g*_2_) = *g*_1_. Now $GeLev\left(G_{1},G_{2}\right)=\frac{0}{g_{1}}=0$.
- Suppose the genomes have different lengths, with *g*_1_ < *g*_2_, but the genes of *G*_1_ all appear in *G*_2_ in exactly the same order. This is a perfect, orderly match. In this case, *Lev*(*G*_1_, *G*_2_) = *g*_2_ − *g*_1_. Additionally, |*g*_1_ − *g*_2_| = *g*_2_ − *g*_1_ and *min*(*g*_1_, *g*_2_) = *g*_1_. Then $GeLev\left(G_{1},G_{2}\right)=\frac{g_{2}-g_{1}-\left(g_{2}-g_{1}\right)}{g_{1}}=0$, which is what we want.
- At the opposite end of the spectrum, consider two genomes to have totally different genes, with say *g*_1_ <= *g*_2_. Then *Lev*(*G*_1_, *G*_2_) = *g*_2_, |*g*_1_ − *g*_2_| = *g*_2_ − *g*_1_, and *min*(*g*_1_, *g*_2_) = *g*_1_. Then $GeLev\left(G_{1},G_{2}\right)=\frac{g_{2}-\left(g_{2}-g_{1}\right)}{g_{1}}=\frac{g_{1}}{g_{1}}=1$, which is now the maximum value.

It may be worth noting that in an experiment like ours, where strains belonging to the same species are compared, the maximum value of GeLev should never be reached. Since the maximum happens only for disjoint sets of genes, a robust number of core genes in a species like *S. epidermidis* will exclude this possibility.

The idea of normalizing the Levenshtein distance has been proposed before. Yujian and Bo [34] defined the Generalized Levenshtein Distance (GLD) metric, with a range between 0 and 1, similar to our case. The main difference between GLD and GeLev is that the former is zero only when applied to two genomes identical in both gene content and order. When two genomes have common genes in the same order, but may have additional unique genes, GeLev will be zero, but GLD will have some positive, non-zero value.

### Jaccard distance

The **Jaccard distance** [35] is a transformation of the coefficient of community. As applied to pangenome analysis, the distance views genomes as sets of genes. If one genome has the set of genes *G*_1_ and another genome the set of genes *G*_2_, then the Jaccard distance between the two genomes is defined as

$$J_{1,2}=1-\frac{|G_{1}\cap G_{2}|}{|G_{1}\cup G_{2}|},$$

where the notation |_| means the cardinality, or the number of elements of the set. The Jaccard distance is a sub-unitary number, 0 ≤ *J*_1,2_ ≤ 1. At the limits, the Jaccard distance has the following meaning: if *J*_1,2_ = 1 then the sets are disjoint, and if *J*_1,2_ = 0 then the sets are identical. Thus, as the Jaccard distance grows, the sets have less overlap. Notice that the Jaccard distance does not consider the order of the elements in sets *G*_1_ and *G*_2_.

The Jaccard distance as defined above has been used, for example, by Liu *et al*. [36] to cluster 5217 *Staphylococcus aureus* genomes. Clustering based on the Jaccard distance correlated with multilocus sequence typing. The authors proposed a new list of housekeeping genes as markers for the classification of species members. The reason for developing a new list is that some of the housekeeping genes previously used for multilocus sequence typing were present in less than 60% of the *S. aureus* strains.

In another study, Yang et al. [37] applied the Jaccard distance to 114 genomes of *Escherichia coli*. They investigated the relationship between gene content and phylotype and features such as biofilm formation and persistence on meat processing equipment.

## Results and discussion

### Relationship between GeLev and Jaccard distances

Our work focused on a collection of 84 *S. epidermidis* complete genomes. Since we excluded plasmids from the analysis, it would be technically more accurate to refer to comparison of chromosomes, not genomes when discussing our results. We therefore use the term “genome” below, with the understanding that it is an approximation that covers most, but not all of the genetic material of a strain.

**Fig 2:**
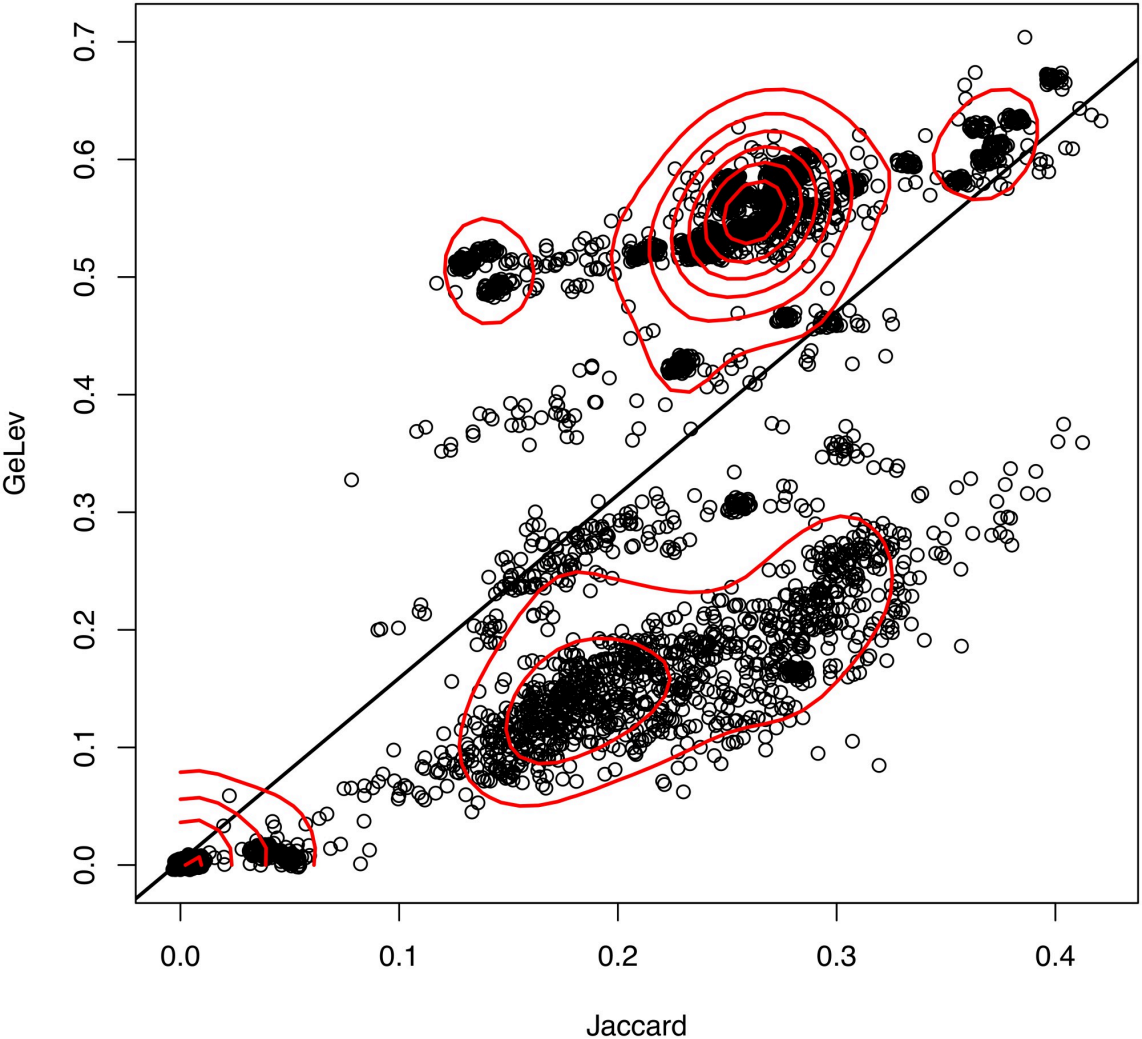
Pairwise comparison of Jaccard distance versus GeLev distance. Each point represents a pair of genomes for which the two distances were computed. Jitter was applied to spread out groups of overlapping points. In red are contour lines that reveal the density distribution of points. The black line shows the dependence of GeLev on Jaccard by simple linear regression.

We first computed all pairwise (84 × 84 = 7056) GeLev and Jaccard distances and plotted them against each other (Fig 2). There were many instances where points for pairs of (*G*_*x*_, *G*_*y*_) genomes did fully overlap. In order to improve the visibility of individual points, the graph used *jittering* to add small random offsets to each point.

The pangenome of the 84 *S. epidermidis* strains had a total number of 7238 genes. The number of core genes that appeared in all genomes was 1587, roughly one third of the total. Therefore, we expected that the Jaccard distance would never be close to 1, as any two genomes would be far from disjoint. In Fig 2, it can be seen that the range of the horizontal axis, which represents the Jaccard distance, is from 0 to roughly 0.5.

The figure shows that the Jaccard and the GeLev distances are not fully correlated, as many points deviate from the line of best fit. The two metrics have different meanings. Points appear to cluster into groups with high within-group correlation. Also noteworthy is that there are several regions of high density, as evidenced by the red contour lines.

Intuitively, the meaning of the two distances as seen in Fig 2 is the following. Consider genomes *G*_1_ and *G*_2_.

**Fig 3:**
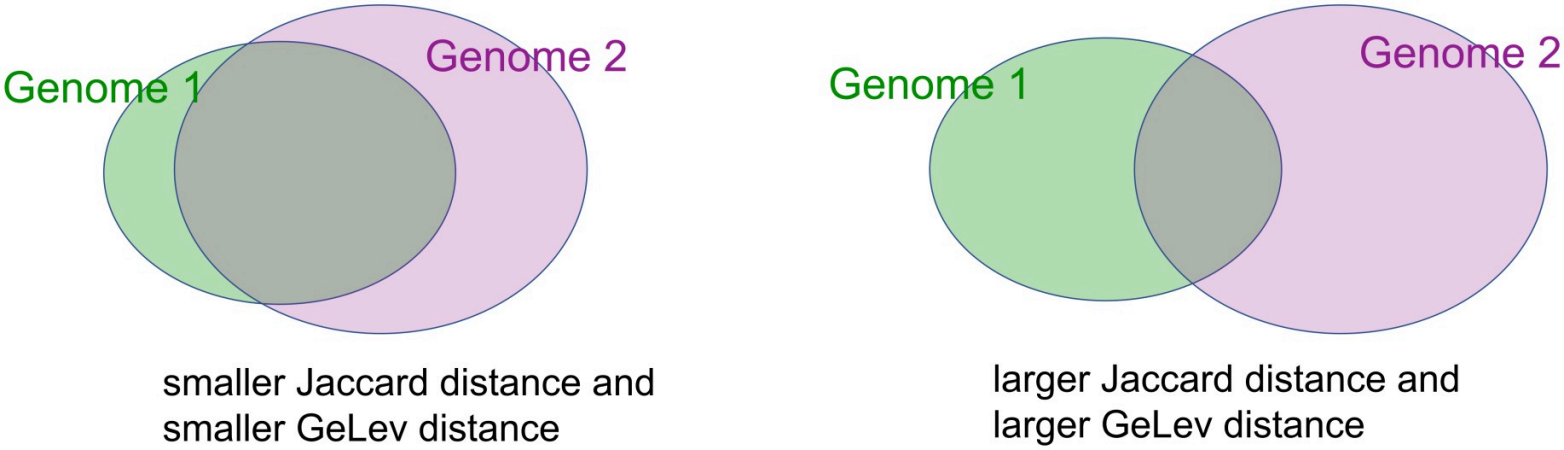
The *cardinality* of the set of common genes affects both the Jaccard and the GeLev distance between two genomes.

**Fig 4:**
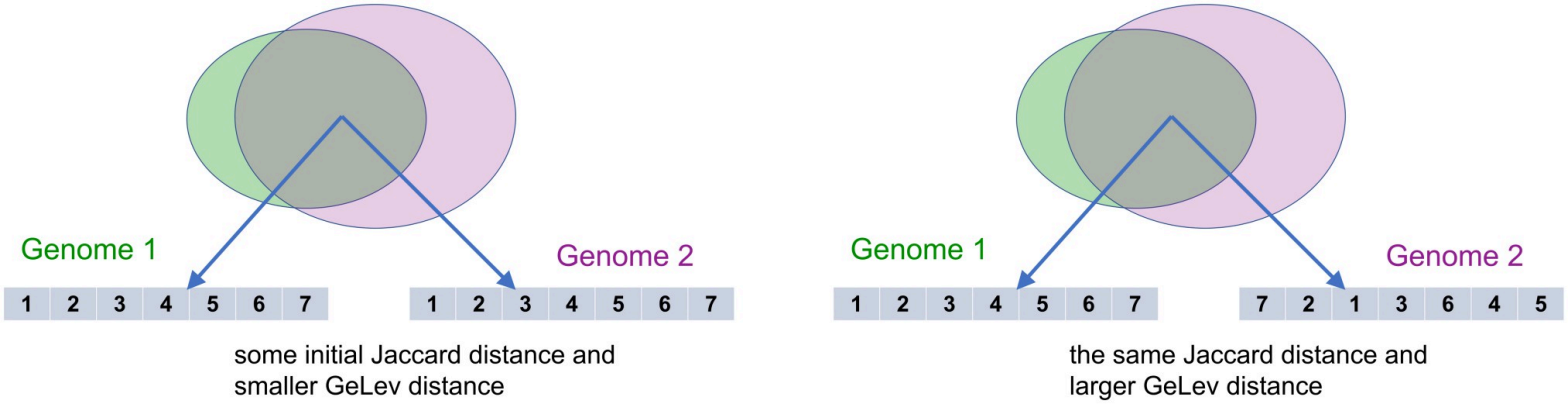
The *order* of the common genes affects only the GeLev distance between two genomes.

- The increase of the Jaccard distance has only one possible cause, namely, there is an increased percentage of genes that do belong only to one of the genomes, but not to both, see Fig 3.
- The increase of the GeLev distance has two possible causes. The first cause is exactly the same as before, there are more genes that are not common to both genomes, see Fig 3. The second cause is that common genes appear in a different order, see Fig 4.

Therefore, the genome pairs of special interest for this research are the ones that measure up differently in the Jaccard distance as opposed to the GeLev distance, namely, that the different measurements in the distances show that the order of the common genes is different.

### Classification of genomes based on GeLev distance

Relationships among the *S. epidermidis* genomes could be better revealed by applying multidimensional scaling (MDS) to the GeLev and Jaccard distance matrices. In this approach genomes are modeled as points that exist in one of GeLev or Jaccard hyperspaces. The exact coordinates of points in the hyperspaces is initially unknown, but they have the property that, for example in the GeLev space, the distance between any pair of genomes (*G*_*x*_, *G*_*y*_) closely matches the *GeLev*(*G*_*x*_, *G*_*y*_) distance. The MDS technique takes as input a distance matrix and computes the coordinates of each point, such that the distances are matched as closely as possible on average. As our distance matrices were of size 84, the spaces produced by MDS were 83-dimensional (84 − 1). MDS also ranks the dimensions by the magnitude of the spread of points, with the first dimension having the largest spread. An eigenvalue associated with each dimension provides a quantitative measure of the variability covered by that dimension. In our case, the first dimension for both GeLev and Jaccard had an eigenvalue by almost an order of magnitude larger than the second. The first 3 dimensions of the spaces covered well over 90% of the variability of the point distribution.

**Fig 5:**
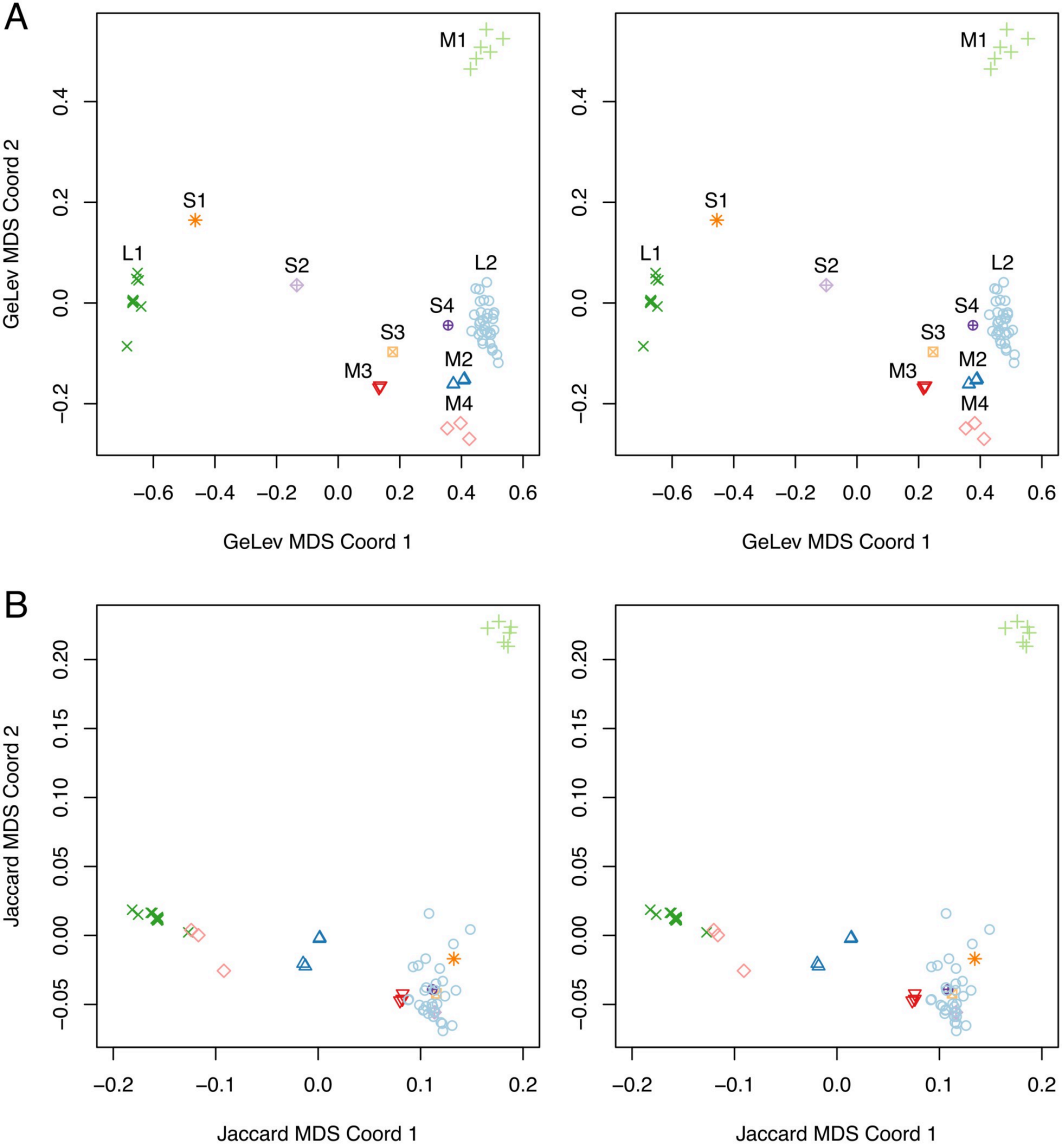
MDS of 84 *S. epidermidis* genomes by GeLev (A) and Jaccard (B) distances, shown as cross-view 3D images. The first and second dimensions are shown on the horizontal and vertical axis, respectively. The third dimension is perpendicular to the plane of the page. Clusters of genomes in the GeLev space are labeled, and their member points distinguished by color and plot symbol.

The layout of genomes in the first 3 dimensions of the GeLev and Jaccard MDS spaces is shown in Fig 5. By visually examining the GeLev plot, we defined 10 different genome clusters, which we labeled based on their size and position: 2 large clusters (**L1** and **L2**) with 32 genomes each, 4 medium clusters (**M1** to **M4**) having between 3 and 6 members, and 4 singleton clusters (**S1** to **S4**). Cluster membership of each strain is shown in Table 1.

It may be tempting to conclude that the larger clusters are more representative of the *S. epidermidis* species and that singletons are outliers. However, the collection of 84 published genomes is not a random sample of the general *S. epidermidis* population. One should expect potentially large biases in sample collection that depend on geography, ecological niche, collection time, and research laboratory. For example 31 of the 32 members of cluster L1 were collected by the same team at a hospital in Saarland, Germany, between 2018-2020 [38]. Nevertheless, examination of the metadata of all genomes did not uncover any other clear correlation of such potential sources of bias with cluster membership. Even in the case of L1, the last sample had a completely different origin, having been collected in Dublin—Ireland in 2013.

The distribution of GeLev clusters in the Jaccard MDS space reveals both similarities and differences between the two spaces (Fig 5B). The three largest clusters, L1, L2, and M1 are compact and far from one another in Jaccard space, similary to GeLev. Other clusters have shifted positions in Jaccard space and most have lost their individuality. For example, M4 is close to L2 in GeLev space, but clusters together with L1 in Jaccard space. Similarly, all singletons are grouped together with L2. Also, M2 has split into two subclusters and is far away from other genomes in Jaccard space. Such mixed behavior is consistent with the observations made from Fig 2 as well as with the theoretical properties that GeLev and Jaccard measure distinct properties of genomes, but are correlated.

### Gene order and structural genome classes

Comparing the relative positions of genomes between GeLev and Jaccard spaces shows that often times they are preserved. However, of particular interest are pairs of genomes (or classes of genomes) that have very different distance values in the two spaces. A high GeLev and low Jaccard distance between two genomes would indicate large differences in gene order of two mostly overlapping gene sets. On the other hand, low GeLev and high Jaccard distances would be produced when two genomes would have different gene sets, but the common genes would be present in the same order.

**Fig 6:**
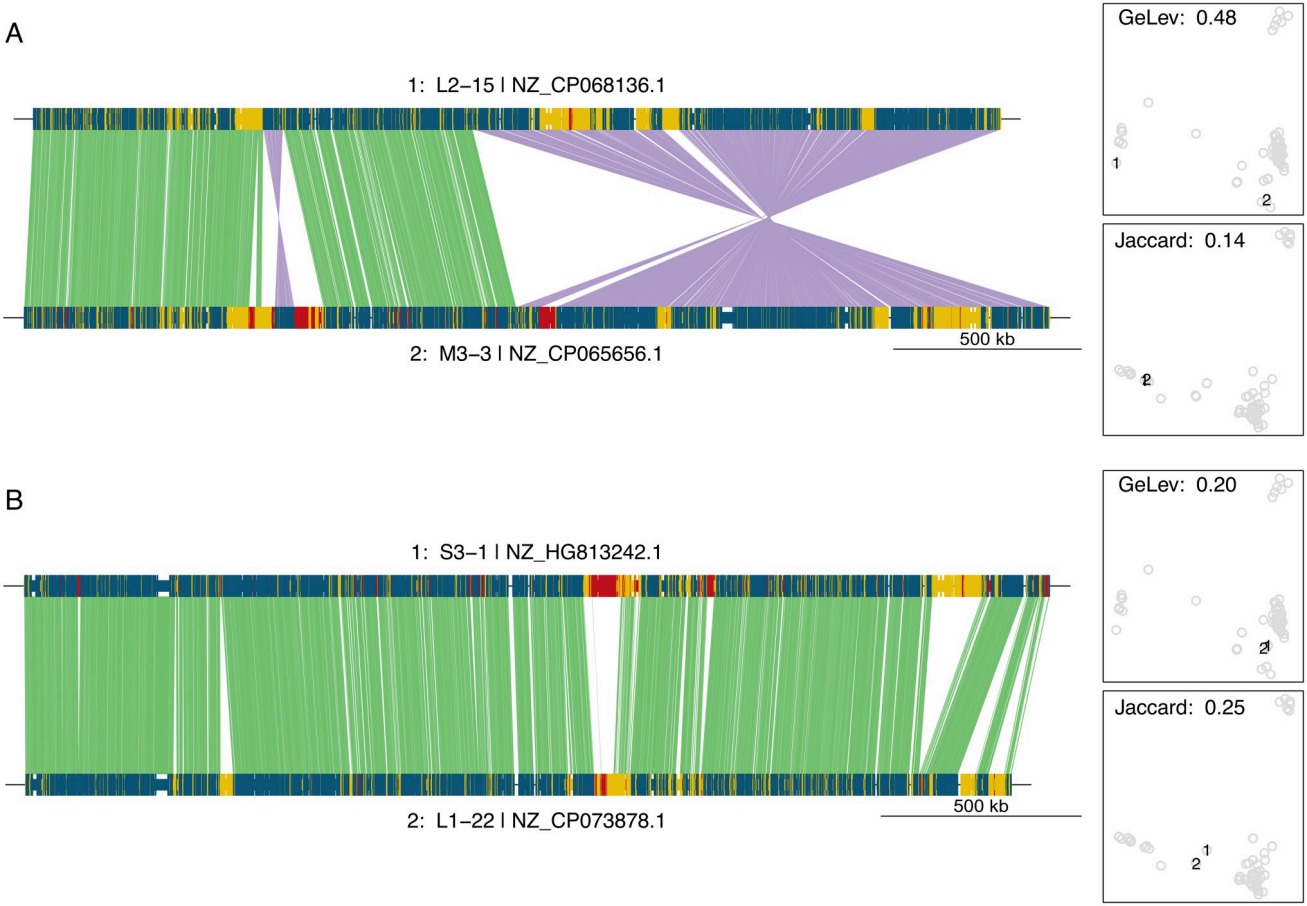
Alignments of genome pairs with contrasting distance values. **A**. A genome pair with high GeLev and low Jaccard distance. **B**. A genome pair with low GeLev and high Jaccard distance. For each pair, the chromosomes are shown as a linear array of genes and are labeled with their cluster identifier, genome number within the cluster, and accession number. Colors indicate core genes, which are present in all genomes (blue), accessory genes present in at least 35% of genomes (yellow), and other, less frequent genes (red). Lines between genomes connect individual genes present in both genomes and oriented in the same (green) or opposite (purple) direction. The panels on the right outline the GeLev and Jaccard spaces as in Fig 5 and have the two genomes under consideration highlighted as 1 and 2.

The structure of genomes that produce contrasting distance values can be better understood by examining alignments of genome pairs. In one extreme example (Fig 6A), the genomes can be conceptually divided into 4 segments, 2 of which are in the same and 2 in the opposite order. Even accessory genes follow the overall order pattern. This explains the unusually high GeLev distance. Gene content, by contrast, is very similar, giving a low Jaccard distance value.

At the other end of the spectrum, Fig 6B shows an example of two genomes with low GeLev and higher Jaccard distance. Common genes are strictly aligned, but significant islands of accessory genes exist, which are present in one, but not the other genome.

We inspected visually all pairwise alignments to uncover gene order differences among clusters and individual genomes. This led to the following observations:

1. Differences in gene order manifested themselves as reversals of large genomic segments, consisting of hundreds of genes. Within a segment, gene order was preserved, i.e. the region defined by a segment has identical synteny in both genomes, only the orientation is reversed. The presence of out of order single genes or small groups of genes was less common than large segment reversals.
2. Within each of the 10 clusters, the genes common to all cluster members were in the same order.
3. The difference between two clusters can be caused by
  (a) differences in gene content, with the shared genes in the same order, and/or
  (b) the presence of reversed segments, irrespective of gene content.

**Fig 7:**
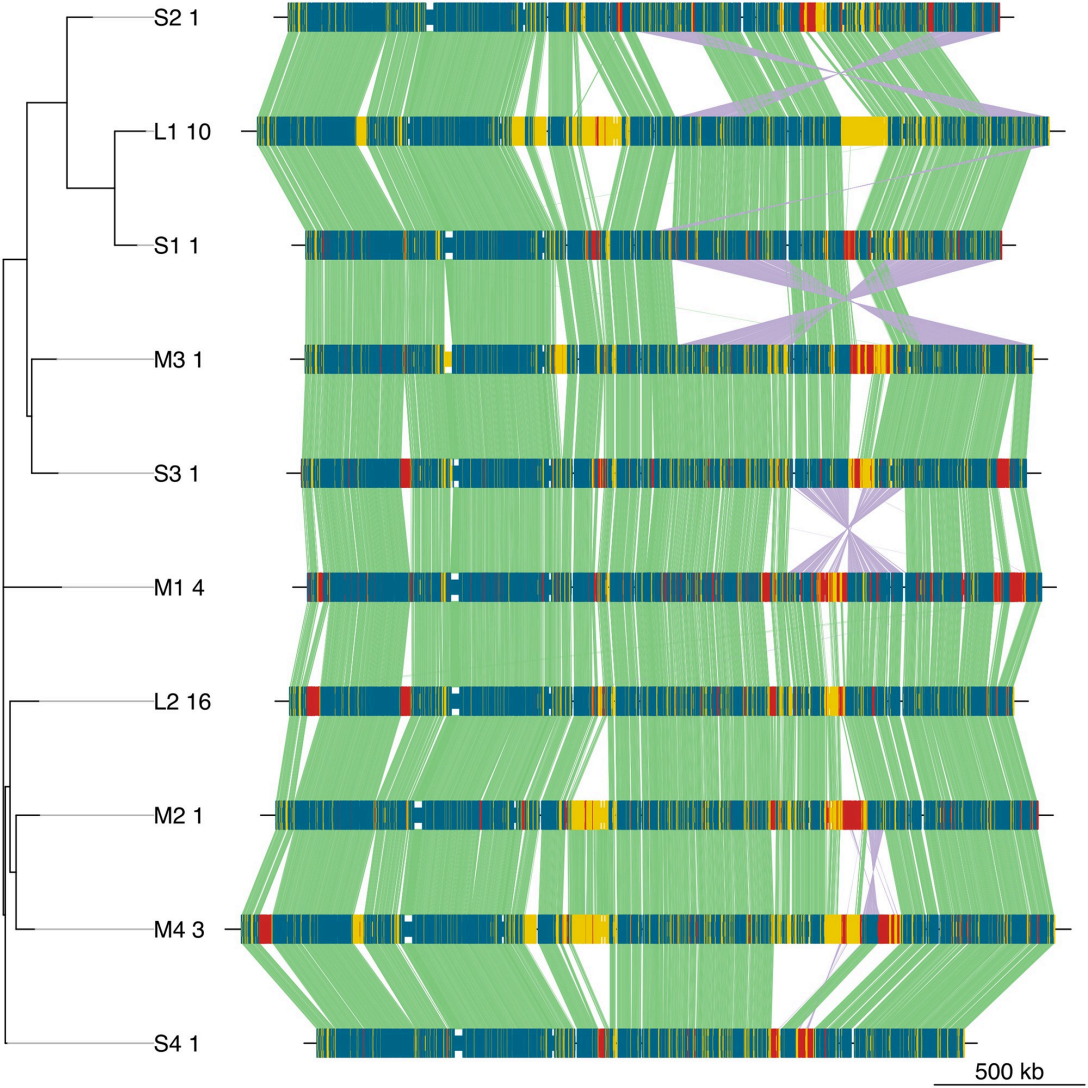
Comparison of the 10 genome structure classes. A representative genome of each GeLev cluster was manually chosen by visual inspection the MDS plot in Fig 5A. The selected genomes were used to construct a multiple alignment based on common genes. The significance of colors and lines is the same as in Fig 6. Each genome is labeled with the cluster identifier and the number of the genome within the cluster (see Table 1). The order of clusters is given by the tree on the left, constructed from GeLev distances.

From these observations it can be concluded that the GeLev distance is able to categorize *S. epidermidis* strains into broad classes, which differ by overall genomic structure. Since genomes within a cluster have common structure, we manually selected a representative from each cluster for illustration purposes (shown in bold in Table 1). For large and medium clusters the representative genome was selected from near the center of the cluster, by visually inspecting the MDS plot in Fig 5A. The relationships among cluster representatives are shown in Fig 7 as a neighbor-joining tree and alignments based on common genes. For example, clusters S3 and M1 differ by the order of a single genomic segment of about 400 kb. In other cases, such as clusters L2 and M2, the gene order is the same, and clusters are distinguished by gene content.

Thus, investigation of gene order in addition to gene content turns out to be a powerful tool for the classification of strains of *S. epidermidis*. The existence of genomic structural classes may have functional significance. Correlations between genetic rearrangements, insertions, and deletions and phenotypic variations in pathogenic and commensal strains have been previously described [39]. As an example, Ziebuhr et al. [40] examined the microevolution of a strain of *S. epidermidis* in a patient with recurring infections associated with a ventriculo-peritoneal shunt. They observed genomic rearrangements and a simultaneous loss of the capacity of the strain to form biofilms. Their data point to the mobile genetic element IS256 as the cause of the rearrangements, suggesting this as a mechanism of adaptation.

### Limitations and future work

Our method has been applied to complete genomes only. This is a limitation of the GeLev metric method. The NCBI Genome database includes four genome assembly levels: contig, scaffold, chromosome, and complete. Our method has been shown to work on complete genomes and may work on chromosome-level assemblies as well. The method definitely does not work on scaffold and contig-level assemblies, which account for over 90% of the NCBI entries for *S. epidermidis*. This means that a very large body of available data is still not taken into consideration. The abundance of incomplete assemblies is attributable to short read sequencing technologies, which have been available since the earliest sequencing efforts. More recent technological advances, such as long read sequencing, is expected to rapidly increase the availability of complete genomes. Therefore, we expect our method to be more readily applicable in the near future, for all species.

Another limitation is given by the output of Roary itself, where some genes are annotated as duplicates, and this annotation may differ from other annotation software. The GeLev algorithm handles duplications organically, without the need of special treatment, as it considers genes as letters, which can occur in a genome string any number of times. Thus, as long as gene annotation is done consistently, the calculated GeLev distance is relevant and depends only on the correctness of the annotation software.

Our definition of genome classes was based on manual clustering, by visual inspection of the MDS plots. We relied on the innate capability of the human eye to integrate information at different scales and found that the proposed clusters correlated perfectly with large-scale genome structure. Of course, this method can only be applied one species (or dataset) at a time. It would be beneficial to automate the process, such that it could be applied to a large number of species. This could be done by using one of the many existing clustering algorithms. The difficulty consists in the fact that the results of automated clustering depend on many factors, including choice of algorithm, tuning of parameters, and deciding on the final number of clusters. As future work, we propose an approach in which we apply the manual process to a small number of different bacterial species, followed by development of an automated pipeline that reproduces the manual results as closely as possible.

## Conclusion

The present research has applied two distance metrics to pairwise compare 84 genomes belonging to *S. epidermidis*. First, the GeLev distance, defined in this paper, is a normalized Levenshtein distance and depends on gene order along the chromosome as well as gene repertoire. Second, the Jaccard distance is based on gene sets and depends exclusively on gene repertoire.

Our observations led to the following conclusions:

1. GeLev applied to *S. epidermidis* clustered the genomes into structural classes, which differed by the orientation of large chromosomal segments, but also by gene content.
2. Genome comparisons based on GeLev offer a better understanding of relationships among strains of the same species than methods based on gene sets alone, with potential functional and phylogenetic implications.
3. The method can be applied to an arbitrary collection of closely related genomes and would be suitable for the characterization of other prokaryotic species.
